# Microbiome and Gestational Diabetes: Interactions with Pregnancy Outcome and Long-Term Infant Health

**DOI:** 10.1155/2021/9994734

**Published:** 2021-11-25

**Authors:** Caterina Neri, Erika Serafino, Maddalena Morlando, Alessandra Familiari

**Affiliations:** ^1^Dipartimento Scienze della Salute della Donna, del Bambino e di Sanità Pubblica, Fondazione Policlinico Universitario “A. Gemelli” IRCCS, Rome, Italy; ^2^Prenatal Diagnosis and High Risk Pregnancy Unit, Department of Woman, Child and of General and Specialized Surgery, University “Luigi Vanvitelli”, Naples, Italy

## Abstract

Microbiota composition is progressively being connected to different physiologic effects, such as glucose metabolism, and also to different pathologies, such as gestational diabetes mellitus (GDM). GDM is a public health concern that affects an important percentage of pregnancies and is correlated with many adverse maternal and neonatal outcomes. An increasing number of studies are showing some connections between specific microbial composition of the gut microbiota and development of GDM and adverse outcomes in mothers and neonates. The aim of this review is to analyze the available data on microbial changes that characterize healthy pregnancies and pregnancies complicated by GDM and to understand the correlation of these changes with adverse maternal outcomes; this review will also discuss the consequences of these maternal gut microbiome alterations on neonatal microbiota composition and neonatal long-term outcomes.

## 1. Introduction

The human microbiome is the wide community of microorganisms that live in and on the human body. It consists of more than 100 trillion cells [1, 2] and contains 27 times more genes than the human genome [3–5]. The microbiome plays an important role in regulating metabolism, immune function, and behavior in humans [6].

The microbiota is represented by the community of microorganisms present on a certain body site, in particular the gastrointestinal tract (also called gut microbiota), the oral cavity, the skin, the lungs, and the genitourinary tract [2].

Until recently, the intrauterine environment was considered to be sterile except in the case of chorioamnionitis related to bacterial infections and usually associated with adverse pregnancy outcomes including preterm birth [7]. However, it is now clear that the placenta has its own “healthy” microbiota which is not necessarily associated with infections. Therefore, a specific microbiota characterizes also the placenta and the amniotic fluid [8], and it is subject to modifications with the progression of the pregnancy [9].

Gestational diabetes mellitus (GDM) is an increasing public health concern that affects approximately 5-20% of pregnancies, and its prevalence is progressively rising [10, 11]. It has been defined as any glucose intolerance with the first onset or recognition during pregnancy [12] and is associated with many adverse maternal and neonatal outcomes, such as preeclampsia, cesarean delivery, macrosomia, shoulder dystocia, and neonatal hypoglycemia [13, 14].

The role of intestinal microbiota in modulating insulin resistance and the body inflammatory response is well known [15, 16]. Therefore, the potential impact of specific interventions on the gut bacteria composition and function is of considerable interest when seeking the optimal strategy to prevent and treat GDM.

The aim of the present work is to review the role of the microbiome during pregnancy, its physiological modifications among trimesters, and its pathological changes when pregnancy is complicated by gestational diabetes. A brief excursus on the molecular approaches to study the gut microbiome will be presented as well. In addition to this, a review of the mechanisms implicated in the correlation between microbiota alterations, adverse pregnancy outcomes, and neonatal long-term outcome will be performed.

## 2. Gut Microbiota Modifications during Pregnancy

With the term “gut microbiota,” we refer to the microorganisms that colonize the gastrointestinal tract [17]. By now, we know that all these microorganisms, which are more than 100 trillion [18] classified in over 35,000 bacterial species [19], have a symbiotic exchange with the human host through the performance of multiple functions [20]: they are involved in nutrients, xenobiotic and drug metabolisms, antimicrobial protection, immunomodulation, integrity of the gut barrier, and structure of the gastrointestinal tract.

The gut microbiota is composed by several types of microorganisms, including bacteria and viruses. Bacteria are classified in phyla, classes, orders, families, genera, and species [21]. The dominant phyla are *Firmicutes* and *Bacteroides*, which represent 90% of the microbiota, followed by *Actinobacteria*, *Proteobacteria*, *Fusobacteria*, and *Verrucomicrobia* [22]. For each phylum, there are predominant genera and species, for example, *Firmicutes* phyla are represented by Clostridium genera for 95% of its composition but includes also other important genera like *Lactobacillus*, *Bacillus*, and *Ruminococcus*. The composition of the gut microbiota changes between individuals and within the same individual in relation to different factors: gestational age at birth, mode of delivery, age, diet, antibiotics, use of probiotics, body mass index (BMI), and exercise are some of the more studied elements that can influence the human gut microbiota composition. Another factor that can influence the microbiota composition is represented by pregnancy, which is characterized by profound hormonal and metabolic changes [21].

### 2.1. Changes of Gut Microbiota throughout Different Trimesters of Gestation

During normal pregnancies, the composition of the gut microbiota changes through the course of gestation: during the first trimester, it resembles that of a healthy nonpregnant individual [9, 23, 24], then it changes gradually, and by the third trimester, it is like the microbiota of people affected by metabolic syndrome, with the capacity to induce it if transplanted in germ-free mice [9]. In particular, the main change is represented by a reduction in alfa-diversity (which is the complexity of species diversity in the sample) and an increase in beta-diversity (which is between-subject diversity) [25]. At the phylum level, there is an increase in *Actinobacteria* and *Proteobacteria* and a decline in butyrate-producing bacteria. Butyrate is an important short-chain fatty acid (SCFA) that can serve as a second messenger as well as a source of energy. These changes might be linked with the maternal metabolic profile, consisting of a decline in insulin sensitivity and an increase in nutrient absorption that are necessary to support a healthy pregnancy [26].

More recent studies have focalized on differences between nonpregnant and pregnant individuals at a genera and species levels, with results that are not always concordant. The genera *Blautia* and *Collinsella* have been shown to increase not only in normal pregnancy [27, 28] but also in pregnancy complicated by GDM [29]; its reduction has been associated with digestive diseases that are common in pregnancy, such as vomit and constipation, and also in disease that is more rare like acute fatty liver; these diseases have also been associated with an increase in the presence of *Paenibacillus*, *Acinetobacter*, and *Enterococci* [25], while in normal pregnancy, there is a reduction in *Acinetobacter* [27].

**Figure 1:**
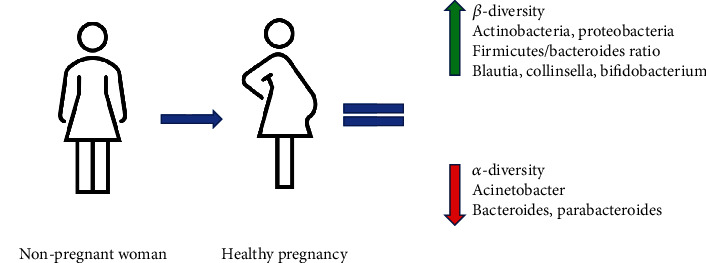
Differences in the gut microbiota between a nonpregnant woman and healthy pregnancy.

Recently, it has been shown that there is a change in the gut microbiota composition from first trimester to second trimester: in particular, there is an increase in *Firmicutes*/*Bacteroides* ratio, *Blautia*, *Rothia*, and *Bilophila* and a decrease in *Bacteroides* and *Parabacteroides* [27] (Figure 1).

Another genera that increases during pregnancy is represented by *Bifidobacterium* with a demonstrated causal role of progesterone in this variation as shown by using murine models with progesterone implanted subcutaneously [28]. *Bifidobacterium* abundance has been shown to be directly correlated with high-fat diet before and during pregnancy, just like *Akkermansia* [30], suggesting a possible role of prepregnancy diet on the type of microbial changes that occur during pregnancy, even if a previous study had shown an inverse correlation between gestational weight gain and reduced abundance of *Bifidobacterium* and *Akkermansia* [31].

Further studies are needed to elucidate the differences between the gut composition of nonpregnant individuals and healthy pregnancies. In consideration of the role played by the gut microbiota in different metabolic processes, research in this field of interest could help understanding the physiology of pregnancy microbiome modifications, and consequently, it could allow developing a strategy of interventions and prevention in high-risk pregnancies.

## 3. Gut Microbiota in Pregnancy: Molecular Approaches

Until recently, information about the microbes inhabiting the human body was obtained via conventional culture-based microbiology techniques, where fluid or epithelial swabs from a given body site were placed in culture media, and the organisms that grow were phenotypically and genetically characterized [32]. Nowadays, Real-Time-q Polymerase Chain Reaction (RT-qPCR), shotgun sequencing of 16S rRNA/rDNA gene sequence, and fluorescent in situ hybridization coupled with flow cytometry are most widely used to characterize the gut microbiome in human and animal models [15].

Animal models have advanced our understanding of the gut microbiome and its relationship to fetal programming. A murine model of parental high-fat diet consumption found that offspring of Western diet breeders had a significantly increased *Firmicutes*-to-*Bacteroides* ratio compared with control offspring and showed heightened colonic inflammatory responses and dysregulated autoimmunity and allergic sensitization [33]. Similar deleterious changes in the maternal and infant microbiome have also been noted in the setting of human studies, advocating the potential protective role of oral administration of probiotic bacteria to pregnant women resulting in colonization of the infant gut lasting from six to 24 months postpartum [34].

Further mechanistic studies, especially in humans, are needed to better understand how gut microbiota interact with the host immune response, especially in the setting of maternal metabolic syndromes, in order to develop targeted interventions during pregnancy and prevent chronic disease in future generations.

## 4. Gut Microbiota in Pregnancies Complicated by GDM

Several studies have shown some differences in microbial composition between healthy pregnancy and pregnancy complicated by GDM, even if not all studies are concordant.

It has been shown that GDM patients have a higher *Firmicutes*/*Bacteroides* ratio when compared with healthy pregnancy patients [23]. The same study found an abundance of *Akkermansia* in the control patients and increased levels of *Lachnospiraceae*, *Phascolarctobacterium*, and *Christensenellaceae* in women with GDM, but no differences at a genera level between the two groups of patients [23].

Some differences were found in the composition of gut microbiota during the third trimester of pregnancy, with the identification of phylum *Actinobacteria* as biomarkers of GDM; in the same study, the genera *Collinsella*, *Rothia*, *Actinomyces*, *Desulfovibrio*, *Leuconostoc*, *Granulicatella*, and *Mogibacterium* were biomarkers of GDM, while the genera *Marvinbryantia*, *Acetivibrio*, and *Anaerosporobacter* were markers of normal glucose regulation [35]. Another small study found that in the third trimester of women with GDM, there is a higher relative abundance of *Bacteroides caccae*, *Bacteroides massiliensis*, and *Bacteroides thetaiotaomicron* and a reduction of *Bacteroides vulgatus*, *Eubacterium eligens*, *Lactobacillus rogosae*, and *Prevotella copri* [36].

**Figure 2:**
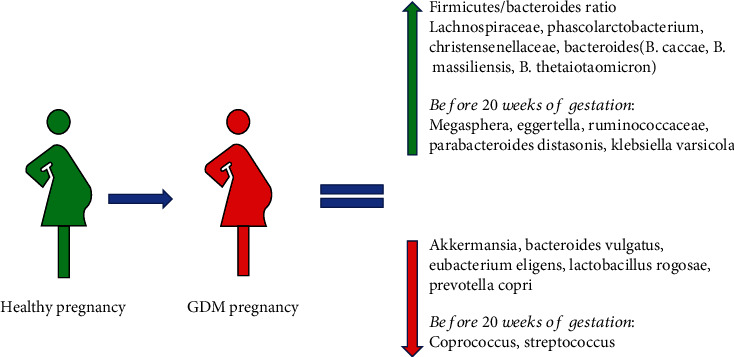
Differences in the gut microbiota between healthy pregnancy and GDM pregnancy.

Differences between gut microbiota in healthy pregnancy compared to pregnancy complicated with GDM are shown in Figure 2.

More recently, several studies have focused on the identification of differences in abundance and composition of the gut microbiota in the first half of pregnancy that correlate to GDM, diagnosed with the standard oral glucose tolerance test at 24-28 weeks of gestation, aimed at discovering an early biomarker for the diagnosis and treatment of gestational diabetes [27, 37–40].

During the first and second trimesters, a decreased relative abundance of *Coprococcus* and *Streptococcus* has been found, which are, respectively, a butyrate-producing bacterium and a lactate-producing bacterium. The same study also showed a positive association between GDM and *Megasphaera* and *Eggertella* [27].

Another study showed, other than a reduced alfa-diversity in patients that will develop GDM, that the genera *Bacteroides*, *Dialister*, and *Campylobacter* were taxonomic biomarkers of GDM, while the genera *Gemminer* and *Bifidobacterium* were markers of normal glucose levels during pregnancy [37]. In contrast with the result of this study, a change in GDM patients from the second to the third trimester has been reported, represented by a higher alfa-diversity, an increment in the colonization of *Firmicutes*, and a reduction in the presence of *Bacteroidetes* and *Actinobacteria* [38].

The increase of relative abundance of *Ruminococcaceae* in the early pregnancy has also been associated with the subsequent development of GDM [39]. In a recent metagenomics study, an association between *Parabacteroides distasonis* and *Klebsiella variicola* in GDM in comparison to healthy pregnancies has been shown [40].

Further studies are needed to understand if interventions on gut microbiota composition in the first half of pregnancy in women with an abundance of microorganisms connected to development of GDM may help prevent the onset of the disease or reduce its severity, with consequent reduction of maternal and neonatal adverse outcomes.

Another group of studies have focused on the identification of specific microorganisms as markers of carbohydrate metabolism. The genera *Blautia* and *Eubacterium hallii* group was positively correlated to fasting blood glucose while the relative abundance of *Faecalibacterium* was negatively correlated to it [38, 41]: the authors suggested the possibility of using these as markers of GDM that is not controlled by diet.

High blood glucose values corresponded to low intestinal *Faecalibacterium/Fusobacterium* ratios, with the correlation highly significant between the bacterial rations and two-hour blood glucose levels, representing the regulatory and recovery capability after sugar intake [42].

Ketonuria, which is an indirect marker of glucose metabolism, has been shown to be associated with a relative abundance of *Roseburia* and also with *Faecalibacterium* and *Dialister* in overweight and obese women at 16 weeks of gestation [43], even if previous studies showed a decrease in *Roseburia intestinalis* and *Faecalibacterium prausnitzii* in patients with type 2 diabetes [44, 45].

Insulin, c-peptide, and HOMA-IR (Homeostatic Model Assessment for Insulin Resistance) have been positively associated with the genus Collinsella in early pregnancy of obese and overweight women [26]; the same study has noted a positive correlation between the genus Coprococcus and the levels of GIP (Gastric Inhibitory Peptide), an incretin that acts by stimulating insulin secretion.

HbA_1c_ levels have been found to be correlated with *Bacteroides* and *Prevotella* [38].

It would be interesting to discover if the use of these biomarkers in the clinical practice may help improve the management of patients with GDM by recognizing patients that are not well controlled with therapy and may need further treatments.

There are some contrasts in the results of some studies, like a recent one [46] that showed no correlation between specific microbial species and GDM in obese and overweight women. The authors linked this result to the use of a more accurate approach, even if the same authors admit that other studies, using the same technique, have found a correlation between GDM and gut microbiota composition [41, 47].

A study that compared the gut microbiome composition between women with a history of GDM and nondiabetic women found no differences after five years from delivery, suggesting that there is no causal role of microbiome composition in GDM appearance [48]; however, another study evidenced a different composition in GDM after eight months from delivery compared to healthy patients, with the genera *Collinsella* and *Olsenella* found to be biomarkers of previous GDM [35].

Further studies are needed to better understand if the differences in gut microbiota composition continue after the term of pregnancies, playing a role in the development of GDM in subsequent pregnancies and if interventions on its composition in the interpregnancies interval may help prevent the onset of GDM.

## 5. Microbiota Alterations in GDM and Adverse Pregnancy Outcome

Over the course of a normal pregnancy, women undergo several physiological changes, including an increase in insulin resistance (IR). In order to compensate for this physiological resistance, insulin secretion increases gradually during gestation [49]. However, some pregnant women have a limited capacity to increase insulin production and, consequently, develop GDM [50]. Dysbiosis, an altered microbiota composition, has been hypothesized to play a key role in the pathogenesis of many acute and chronic conditions, including metabolic diseases, such as obesity, insulin resistance, and both type 1 and type 2 diabetes mellitus (T2DM) [51, 52].

The composition of the microbiome changes during pregnancy. It has recently been proposed that intestinal microflora and their metabolic activities (intestinal dysbiosis) may play a critical role in body weight control, energy homeostasis, fermentation, and absorption of nondigestible carbohydrate and also in the development of IR; therefore, it may also participate in the pathogenesis of several metabolic disorders, such as obesity, diabetes mellitus, and GDM [50, 53].

In addition to the gut microbiome, the composition of the microbial community in other body sites seems to also be involved in systemic health [54–56]. The oral microbiome seems to play an important role in obesity and diabetes, through the release of inflammatory mediators that may increase the IR, suggesting a link between pathogenic periodontal bacteria (such as *Porphyromonas gingivalis* and *Aggregatibacter actinomycetemcomitans*) with glycemic control and risk of diabetes [54].

During pregnancy, there is a change in the structure of the vaginal bacterial community, leading to the production of metabolites such as lactic acid that helps to maintain low pH, which contribute to increasing the presence and stabilization of Lactobacillus in the vaginal microbiome. New data concerning the relationship between the vaginal microbiome and metabolic diseases, such as GDM, have been reported [57]. An increase of inflammatory cytokine expression has been shown in GDM, as well as an increase in the abundance of potential pathogenic bacteria, characterizing a dysbiotic profile of the vaginal microbiome [23, 57].

Currently, the etiology is unknown for some of the most important obstetric conditions, such as preeclampsia, premature preterm rupture of membranes, premature labor, preterm delivery, intrauterine growth restriction, gestational diabetes, abruptio placentae, late abortions, stillbirth, hyperemesis gravidarum, and gestational trophoblastic disease, although a microbial role has been implicated in all these conditions. In a recent publication, Romero coined the term “The great obstetrical syndromes” [58] referring to syndromes characterized by multiple etiologies, long preclinical stage, frequent fetal involvement, often adaptive clinical manifestations, and predisposing genetic interactions. Diagnosis and treatment for any of these conditions is challenging, although changes in the microbiota were suggested to play a role [59].

### 5.1. Cardiometabolic Adverse Outcome

There is mounting evidence supporting the role of the gut microbiome in cardiometabolic diseases in pregnancies [60, 61], and the imbalance in the gut microbiome is nowadays considered an important contribution to the development of GDM [61, 62] being already demonstrated that differences in gut microbiome composition, and its related metabolic activities, distinguish lean versus obese individuals and those with type 2 diabetes mellitus versus those without. Moreover, the finding that a different microbial pattern precedes the onset of GDM leads to the hypothesis that microbiota alterations might have a role in the pathogenesis of GDM [9, 39].

More difficult is the topic over the relationship between the composition of the microbiome in pregnancies complicated by GDM and adverse obstetrical outcomes.

### 5.2. Preterm Birth

Actually, the proof of a link between alteration of the microbiome in pregnancy and adverse obstetrical outcomes is various: a review of the literature [63] and a meta-analysis of 22 studies including 12,047 pregnant women showed that women with periodontitis had an increased risk of preterm delivery (PTD) and of delivering a low-birth-weight infant [64].

### 5.3. Gestational Hypertension, Preeclampsia, and Instrumental Delivery

A dysbiotic microbiome is implicated in the diffusion of gut bacterial endotoxin into systemic circulation, inducing a low-grade inflammatory response, which is a common feature of cardiometabolic diseases and that in turn raises the risk of maternal complications of pregnancies. Combined with insulin resistance, chronic subclinical inflammation characterizes the hallmark pathway to the development of both gestational diabetes and gestational hypertension [65]. The maternal oral, vaginal, and gut microbiome influence the risk of pregnancy outcomes and have profound impacts upon the health of the neonate and infant, potentially affecting the possibility that patients affected by GDM—given the microbiome imbalance—can be super exposed to preterm birth, preeclampsia, and excessive gestational weight gain.

The alteration of the microbiome associated with GDM may contribute to the elevated risk of pregnancy complications, including preeclampsia and instrumental or operative delivery for the mother. Fetal complications include macrosomia (birthweight greater than 4500 g), polyhydramnios, preterm birth, shoulder dystocia, and neonatal complications of admission to high-level care, respiratory distress, hypoglycemia, and jaundice. Both women with GDM and their infants are also at increased risk of diabetes mellitus and metabolic dysfunction later in life [66, 67], and this risk can be connected to the favorable outcome of subsequent pregnancies.

Although incompletely elucidated, there are a number of modifiable factors that shape the composition of the maternal microbiome, including maternal diet, prepregnancy weight and gestational weight gain, and hygiene practices. The maternal microbiome and perinatal factors establish the fetal and infant microbiome.

Indeed, treatment of GDM improves pregnancy outcomes with significant reductions in the rate of serious perinatal outcomes including macrosomia, shoulder dystocia, and caesarean delivery [68, 69]. Primary prevention of GDM rather than treatment would however be ideal in preventing both the economic and health costs associated with GDM.

As a strategy for reducing the risk of adverse pregnancy outcomes that negatively impact neonatal and infant health, practitioners should evaluate women's attainment of a healthy maternal microbiome before and during pregnancy (via preconception and prenatal care) through the promotion of a healthy diet, achievement of a healthy weight status and weight gain during pregnancy, and oral hygiene (such as regular brushing, flossing, and dental care). In the perinatal period, the key target for promoting a healthy infant microbiome includes the promotion of breastfeeding and kangaroo care along with the judicious use and appropriate selection of antibiotics.

There is a need for research to further elucidate maternal microbiome patterns that protect against and elevate the risk for adverse pregnancy outcomes that impact neonatal and infant health and, thereafter, to identify modifiable factors that influence the composition of the maternal and infant microbiome to support the targeting of health strategies to improve pregnancy outcomes and infant health.

## 6. Neonatal Microbiota in Pregnancies Complicated by GDM and Neonatal Long-Term Outcome

It is well known that the maternal environment affects the offspring health. The newborn gut microbiota is strongly influenced by maternal health and pregnancy conditions and participates in the development programming of the newborns [70–72]. Early disruption of the infant microbiota has been associated with many inflammatory, immune-mediated, allergic, and dysmetabolic diseases in later life [70–73].

GDM was found to be associated with specific changes in the gut microbiota composition [23, 35, 38, 40, 42]. The altered microbiome may have a crucial role in the underlying metabolic dysregulation that underpins the pathogenesis of gestational hyperglycemia, as well as the consequence of the increased adiposity frequently coexisting in GDM patients [74, 75].

### 6.1. Neonatal Microbiota

A possible vertical mother-to-child transmission of maternal gut bacteria has already been reported, even if, to date, certainty about the way of intrauterine microbial acquisition is lacking [76–78]. Besides breastfeeding and vaginal microbiota, placenta and amniotic fluid have also been reported to be a vehicle for this transmission [9].

Human and animal studies investigating possible causal linkage of disease programming suggest that gut microbiota dysbiosis negatively affects metabolic health triggering cardiometabolic disease onset later in life [79]. In alignment with the “developmental origin of health and disease” hypothesis, increasing evidence supports that exposure to prenatal metabolic disorders during fetal growth may contribute to health outcomes in the offspring [80].

Among full-term infants, gut microbiota consists primarily of anaerobic organisms. The “normal” infant gut microbiota develops by the colonization of facultative anaerobic organisms, later developing obligate anaerobes, including *Bifidobacterium*, *Bacteroides*, and *Clostridium* [81]. These anaerobes are associated with producing polysaccharides that mediate microbiota colonization, immune modulation, and host-gut cross-talk [70]. For example, *Clostridium* in the infant's gut, at high levels, is pathogenic and considered unhealthy.

### 6.2. Childhood Microbiota

After the age of 3 years, the microbial environment changes rapidly; compositional stability occurs to resemble an adult becoming dominated by *Firmicutes* and *Bacteroidetes* [82].

Gut microbiota is associated with metabolic and immune-inflammatory axes in the liver, muscle, and brain through host pathways. Dysbiosis, or imbalance of the infant gut microbiome, may be facilitated by early exposure to environmental factors such as bacteria and viruses, which can also alter host microbiota. This dysbiosis of microbiota has long-term effects on host metabolism, leading to metabolic changes, in particular, type 1 diabetes, autoimmune disease, and obesity [70]. In humans, it is suggested that early microbial patterns may predict excessive weight gain in offspring during childhood and later in life [70, 83] and that microbiota-related epigenetic changes during early development can affect phenotypic characteristics such as obesity later in life [83]. All these data support the hypothesis that the infant's early exposure to maternal microbiomes through a transfer of maternal gut microbiota may alter the composition of the infant's gut microbiome.

### 6.3. Long-Term Health Status

Recent research reported that GDM alters the microbiota of newborns, contributing to the current understanding of intergenerational obesity and diabetes prevalence [41]. In particular, one study observed a significant reduction in the diversity of various bacterial types in GDM newborns indicating that there might be serious dysbiosis in the gut of GDM newborns [84]. Compared with those of healthy newborns, GDM newborns could be more predisposed to develop gastrointestinal diseases and metabolic syndrome at later stages in their lives [84]. These findings are consistent with previous data showing that the gut microbiota in the GDM group was associated with a lower alpha-diversity level compared with that in the healthy groups [46] which, in turn, is associated with a higher BMI [85]. Research supports that the future health of infants may be affected as the offspring of GDM mothers is more likely to develop obesity during childhood and later in life [79], and this is information that deserves to be included in the prenatal counselling of patients affected by GDM.

Future studies are needed to improve our current knowledge in terms of infant gut microbiome and weight management interventions, important for decreasing risks for obesity and cardiometabolic disorders. Studies that connect diet, microbiota, and metabolism in mothers with GDM and their offspring remain a critical key point in obstetrics research. Further work is needed to determine specific mechanisms of compositional changes in newborns and infants over time.

Finally, efforts to identify biomarkers that detect neonatal dysbiosis are required to define appropriate diagnostic approaches and design effective early intervention strategies to optimize infancy, childhood, and adult health outcomes.

## 7. Clinical Implications

It is clear from the literature published in this field the crucial role of proper maternal nutrition throughout pregnancy in order to maintain a balanced microbiota colonization, which is demonstrated to positively influence intrauterine and vaginal environment, thus leading to reduced risk of both maternal and neonatal metabolic dysfunction and preterm birth.

According to this, probiotics administered during pregnancy are supposed to be helpful in preventing complications such as gestational diabetes. Several studies, investigating the possible role of probiotic use versus placebo in overweight and obese pregnant women, suggest that this can be a valid proposal of prevention strategy in order to reduce maternal and neonatal complications related to dysbiosis [86, 87].

In this scenario, the use of antibiotics during pregnancy should be extremely well weighted, considering risks and benefits of treating mothers with drugs potentially harmful for the microbiota composition.

It is demonstrated that antibiotics may alter the gut microbiota, in terms of the total number of bacteria and also its composition [88, 89]. Whether this change may improve or worsen the risk of developing GDM is yet to be demonstrated. While in nonpregnant individuals it has been shown that antibiotic exposure may increase the risk of type 2 diabetes [90], a retrospective study on 12,551 patients found no differences in the risk of GDM between pregnant women that used antibiotics during pregnancy versus women who did not [91]. Another study found that antibiotic treatment in adolescent mice reduced *Bacteroidetes* [92]. In contrast to this, as it has already been discussed before, *Bacteroidetes*, along with *Dialister* and *Campylobacter*, is considered taxonomic biomarkers of GDM [37]. So, the real impact of antibiotics on the risk of developing GDM is far to be demonstrated.

Future research should focus on demonstrating the usefulness of “mapping” the maternal microbiome early during pregnancy as a preventive strategy to detect and treat unbalanced microbiota colonization that can be later related to adverse maternal and neonatal outcomes.

## 8. Conclusions

It is clear that the maternal microbiome widely influences neonatal and infant microbiome, and it has been shown that microbiome pathological alterations occurring during pregnancy can lead to adverse pregnancy outcomes that negatively affect neonatal and infant long-term health status, with a consistent socioeconomic impact as well.

Further characterization of the maternal microbiome and identification of various factors that facilitate changes in microbial profiles during preconception and in the course of pregnancy may elucidate preconception and prenatal strategies for improving pregnancy outcomes and, thereby, neonatal and infant health.
